# Stimulation strategies for electrical and magnetic modulation of cells and tissues

**DOI:** 10.1186/s13619-023-00165-8

**Published:** 2023-07-01

**Authors:** Suleyman A. Omer, Kaitlyn H. McKnight, Lucas I. Young, Shang Song

**Affiliations:** 1 Department of Biomedical Engineering, The University of Arizona, Tucson, AZ USA; 2 Departments of Neuroscience GIDP, Materials Science and Engineering, BIO5 Institute, The University of Arizona, Tucson, AZ USA

**Keywords:** Stimulation strategy, Electrical modulation, Stem cells, Neural recovery, Musculoskeletal regeneration, Conductive, Piezoelectric, Magnetic, Tissue engineering, Regenerative medicine

## Abstract

Electrical phenomena play an important role in numerous biological processes including cellular signaling, early embryogenesis, tissue repair and remodeling, and growth of organisms. Electrical and magnetic effects have been studied on a variety of stimulation strategies and cell types regarding cellular functions and disease treatments. In this review, we discuss recent advances in using three different stimulation strategies, namely electrical stimulation via conductive and piezoelectric materials as well as magnetic stimulation via magnetic materials, to modulate cell and tissue properties. These three strategies offer distinct stimulation routes given specific material characteristics. This review will evaluate material properties and biological response for these stimulation strategies with respect to their potential applications in neural and musculoskeletal research.

## Background

Electrical signals are essential for the communication of neurons to other cells (Galarreta and Hestrin 2001; Laughlin and Sejnowski 2003) and play an important role in neuronal survival, differentiation, and functional expression (McCaig et al. 2005; Zhang and Poo 2001). Electrical signals are also fundamental in biological processes such as embryogenesis, tissue repair and remodeling, and growth of organisms (Patel and Poo 1982; Prabhakaran et al. 2011; Stewart et al. 2015; Zhang and Poo 2001). Particularly, induced electric fields have been shown to change cellular activities and offer insights in disease mitigation (Henrich-Noack et al. 2017; Priori 2003; Voroslakos et al. 2018). Past research identified naturally occurring electrical currents (average field strength of ∼3 V/m) along the rostral migration path, which could guide neuroblast migration from the subventricular zone in the adult mouse brain (Cao et al. 2013). Exogenous electrical stimulation is equally effective in promoting cell mobilization and differentiation in a voltage- and time-dependent manner (Chang et al. 2011; Heo et al. 2011; Kotnik and Miklavcic 2006; Sato et al. 2009; Zhang et al. 2011). Recently, our studies developed implantable conductive platforms with varying physical shapes to understand molecular and cellular changes affected by electrical effects (Song et al. 2019; Song and George 2017). We further used conductive platforms to actively apply exogenous electrical stimulation on transplanted human neural progenitor cells to treat peripheral nerve injury and stroke in animal models (Oh et al. 2022; Song et al. 2021). The aspect of combining cell therapy with electrical modulation could lead to new ways for successful tissue regeneration and regenerative medicine.

This review focuses on recent advances in stimulation strategies used for electrical and magnetic modulation of cells and tissues for potential organ repair and regeneration within the last decade. The review will describe three distinct stimulation strategies including active electrical stimulation via conductive materials, mechanically induced electrical stimulation by piezoelectrical materials, and magnetic-field induced stimulation to regulate cellular response. We will discuss material properties and biological response from these stimulation strategies for a better understanding of their applications involved in neural and musculoskeletal research.

## Active electrical stimulation via conductive materials

**Fig. 1:**
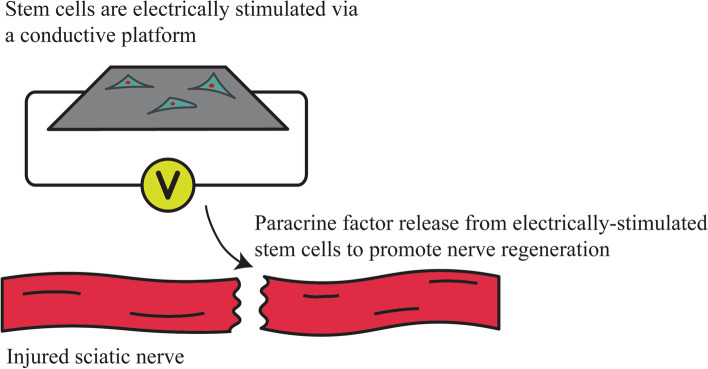
Stem cells are electrically stimulated via conductive material (grey) by an externally applied electrical field. Electrical stimulation results in paracrine factor release from stem cells which promoted nerve regeneration and functional recovery (Song et al. 2021)

Electrical stimulation can be directly applied to cells and tissues through conductive materials to promote desired biological response for tissue regeneration (Fig. 1). Common conductive materials include polymers such as polypyrrole (PPy), polyaniline (PANI), and poly(3,4-ethylenedioxythiophene) (PEDOT) with overlapping pi-bonds that allow for free movement of electrons. Particularly, PPy is one of the most recognized conductive polymers for its high conductivity and excellent biocompatibility (George et al. 2017, 2005, 2009; Oh et al. 2018). Biodegradable conductive hydrogels are also attractive due to their tunable material characteristics such as degradation in addition to electrical properties (Nguyen et al. 2014; Ribeiro et al. 2015). Use of electrical stimulation through implanted conductive materials can be challenging due to the requirement of power sources and device designs (Ben Amar et al. 2015). Recent studies have investigated the effects of using active electrical stimulation via conductive materials for biomedical applications (Table 1).

**Table 1:** Applications for active electrical stimulation

Application	Material	Stimulation Parameters	Results	Ref
Neural	mPEG-PLV grafted with tetraniline	square wave, 10 Hz, 3 mA, 0.1 Volts for 1 h every day for three days	The in vivo study showed thermo-sensitive polymer electroactive hydrogel scaffold promoted spinal cord tissue repair with biochemical cues	Liu et al. 2021a, b
Neural	2D PPy conductive scaffold	800 mV, 100 Hz for 1 h for 3 consecutive days	The in vivo study found that the conductive polymer system enabled electrical modulations of stem cells to improve stroke recovery	Oh et al. 2022
Neural	3D Conductive nerve guide made of PPy	40 V/m at 100 Hz for 1 h (in vitro), day 1,3,5 post implantation (in vivo)	Animal studies showed that electrically-enhanced stem cell based therapy promoted peripheral nerve regeneration and functional recovery	Song et al. 2021
Neural	Collagen/hyaluronan hydrogel with PPy	100 mV/cm 1 h/day for 3 days	The scaffolds supported neuronal differentiation of bone marrow derived mesenchymal stem cells (MSCs) to treat spinal cord injury	Wu et al. 2021
Neural	Electroplating of 2D and 3D PPy scaffolds	40 V/m for an hour	The applied electrical field dictated neural stem cell properties depending on physical nature of stimulating platforms	Song et al. 2019
Bone	Anionic nanofibrillar cellulose (aNFC) hydrogel	0.1 V/cm, 0.04 ms, 10 Hz for 30 min per day	Increased expression of the osteogenic markers (e.g. osteopontin and osteocalcin) and rearrangement and alignment of the actin cytoskeleton inadipose-derived stem cells	Bicer et al. 2020
Cartilage	Hyaluronic acid and gelatin mixture	10 mV/cm at 60 kHz, 30 min, 4 times a day	Higher expression of collagen type II, glycosaminoglycans, and collagen, in MSCs	Vaca-Gonzalez et al. 2020

### Neural applications

Conductive hydrogels are highly attractive because the 3D networks of hydrogels mimic high water content and porous structure in the tissues, facilitating transport of nutrients and oxygen. A previous study reported a thermosensitive conductive hydrogel made of a poly(ethylene glycol)-co-polyvaline (mPEG-PLV) polymer grafted with tetraniline (Liu et al. 2021b). The aniline tetramer segment maintained excellent electrical activity even in an aqueous solution. The authors found that the hydrogel combined with nerve growth factor (NGF) and electrical stimulation (e.g. square wave, 10 Hz, 3 mA, 0.1 V for 1 h daily) substantially increased neurite outgrowth in PC12 cells, which is an immortalized cell line from rat pheochromocytoma. This finding was observed in rats with spinal cord injury, in which implanted hydrogel combined with NGF and transcutaneous electrical treatment significantly improved endogenous neurogenesis and restored locomotor function. A methacrylate anhydride-modified collagen–PPy nanoparticle hybrid hydrogel was constructed to provide a neuroprotective and neuroinductive niche to support neuronal differentiation of bone marrow derived mesenchymal stem cells (MSCs) for treating spinal cord injury (Wu et al. 2021). Although MSCs showed no changes in viability under active electrical stimulation (e.g. 100 mV/cm for one hour daily), they expressed more neuronal markers such as Tuj1 and PSD95, while the glial expression was downregulated. The study found an upregulated expression of L-type voltage-gated calcium channel, suggesting that electrical stimulation activated calcium dependent pathways to enhance neuronal differentiation (Zhu et al. 2019). Implantation of the hydrogel without active electrical stimulation provided short-term protection to transplanted MSCs and increased locomotor function in rats with spinal cord injury.

The use of active electrical stimulation to enhance cell therapy for treating neurological diseases was further demonstrated in our research. A planar, PPy-based conductive system was developed to deliver neural progenitor cells (NPCs) with exogenous electrical stimulation (800 mV, 100 Hz, for 1 h daily) to treat stroke in animals (Oh et al. 2022). We found that post-operative electrical stimulation via the conductive system modulated stanniocalcin 2 (STC2) expression in transplanted NPCs, which augmented the brain’s intrinsic mechanisms of repair such as endogenous stem cell production to enhance restoration following ischemia. Improved functional recovery including vibrissae-forepaw test and beam walking was observed as early as three weeks post-stroke using this regenerative approach. We also fabricated a cylindrical PPy-based conductive nerve guides to treat peripheral nerve injury in rats (Song et al. 2021). Externally applied electrical stimulation (40 V/m at 100 Hz for 1 h daily) through the conductive nerve guide increased neurotrophic factor released from transplanted NPCs, promoting nerve regeneration and functional recovery. The regenerated nerves showed a higher density of axonal fibers and thicker myelination sheath along with increased nerve conduction after 12 weeks of implantation. Accelerated functional recovery was demonstrated in both sensory and motor tests within 1–2 weeks after combined treatment of stem cell and electrical stimulation. The elevated expression of tyrosine kinase receptors (Trk) receptors, which are known to bind to neurotrophic factors, suggested a positive long-term effect from electrical stimulation on peripheral nerve recovery. Other animal studies also showed enhanced expression of regeneration-associated genes and neurotrophic factors in promoting rat peripheral nerve regeneration (Al-Majed et al. 2000a, 2000b). Immediate electrical stimulation following transection and denervation of peripheral nerves exhibited accelerated reinnervation (Gordon 2016). Despite the experiments being conducted using stainless probes rather than conductive polymers, relevant conclusions can still be drawn about the role active electrical stimulation plays in peripheral nerve regeneration. These studies demonstrate that active electrical stimulation via conductive materials can be used to modulate cellular microenvironment and shape the healing process for neurological diseases.

### Musculoskeletal applications

Active electrical stimulation provides external stimuli to enhance cell properties such as differentiation to promote osteogenesis and chronogenesis. Adipose-derived stem cells (ADSCs) embedded within anionic nanofibrillar cellulose (aNFC) hydrogels were treated with electrical stimulation (0.1 V/cm, 10 Hz for 30 min daily) to investigate the osteoinductive potential (Bicer et al. 2020). Electrical stimulation increased expression of osteogenic markers such as osteopontin and osteocalcin and deposited mineralization in ADSCs in 3D hydrogel compared to 2D culture. The positive effect of electrical stimulation on 3D cell culture was also seen in our study. We reported that electrical stimulation on hNPC embedded in cylindrical conductive platform was more effective in enhancing neurotrophic factor expression, whereas those cultured on planar conductive surface showed increased gene expression related to cell–cell adhesion, neuronal differentiation, and metabolic maintenance (Song et al. 2019). Electrical stimulation of MSCs encapsulated in hyaluronic acid (HA)-gelatin (GEL) mixture was studied for the chondrogenic potential of the hydrogel (Vaca-Gonzalez et al. 2020). MSCs exposed to electrical stimulation (10 mV/cm, 60 kHz for 30 min every 6 h a day) were rounder in morphology and reported a higher level of chondrogenic markers such as SOX-9 and aggrecan compared to unstimulated groups. Electrically stimulated MSC-hydrogel also showed glycosaminoglycans and collagen content due to elevated chondrogenic expression. These studies demonstrate that the dimensionality of cell culture as well as electrical platform, plays a critical role in dictating cell functions.

## Mechanically induced stimulation by piezoelectrical materials

**Fig. 2:**
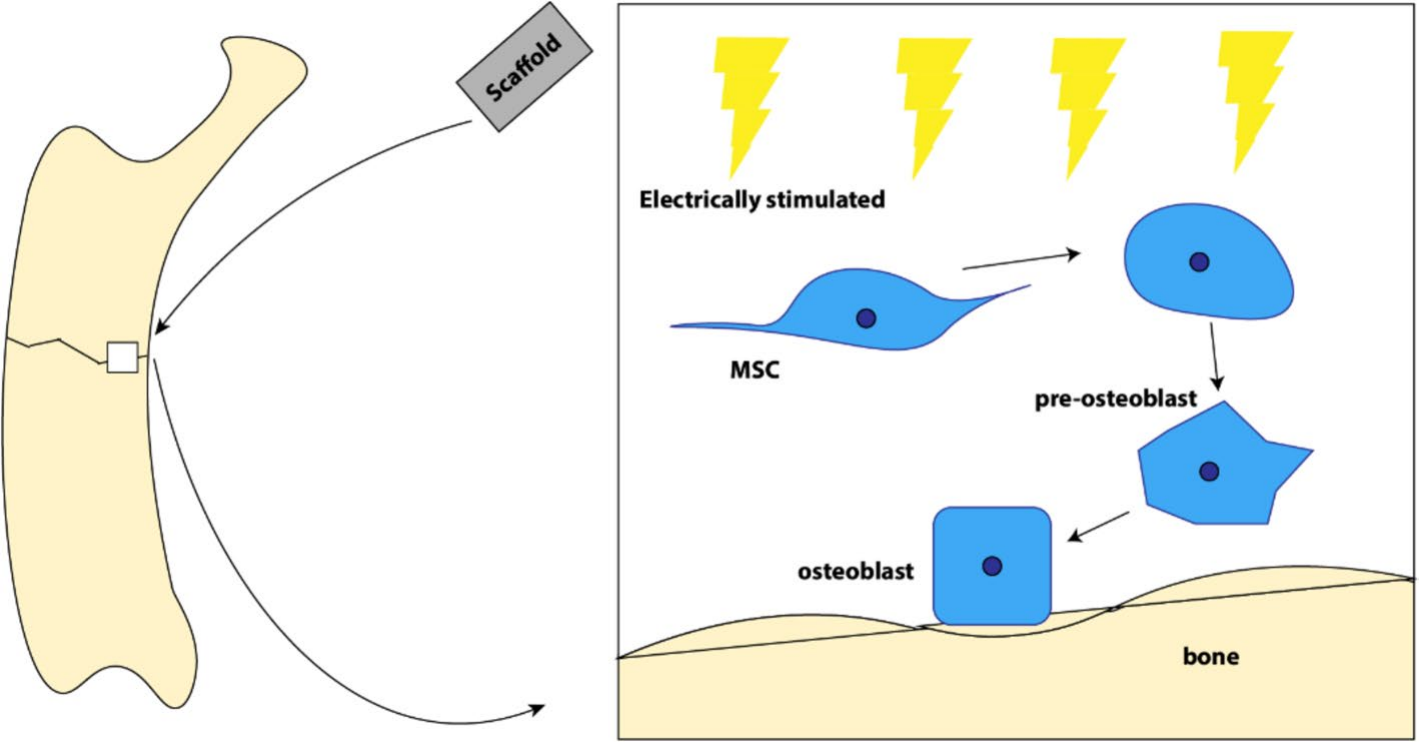
Bone remodeling process in response to piezoelectric stimulation. Damage to the bone is treated with a piezoelectric scaffold placed within the site of impact. The physical interaction of the scaffold and bone defect polarizes that space. Electrical signals produced by the piezoelectric scaffold cause mesenchymal stem cells (MSCs) undergo cell differentiation, advancing to a stage of pre-osteoblasts. After further maturation, the original MSCs complete differentiation into osteoblasts (D'Alessandro et al. 2021). Osteoblasts produce bone matrix and integrate with the host tissue. This process of cell differentiation that initiates the process of bone remodeling is induced by the piezoelectric signals from the scaffold (Leppik et al. 2020)

**Fig. 3:**
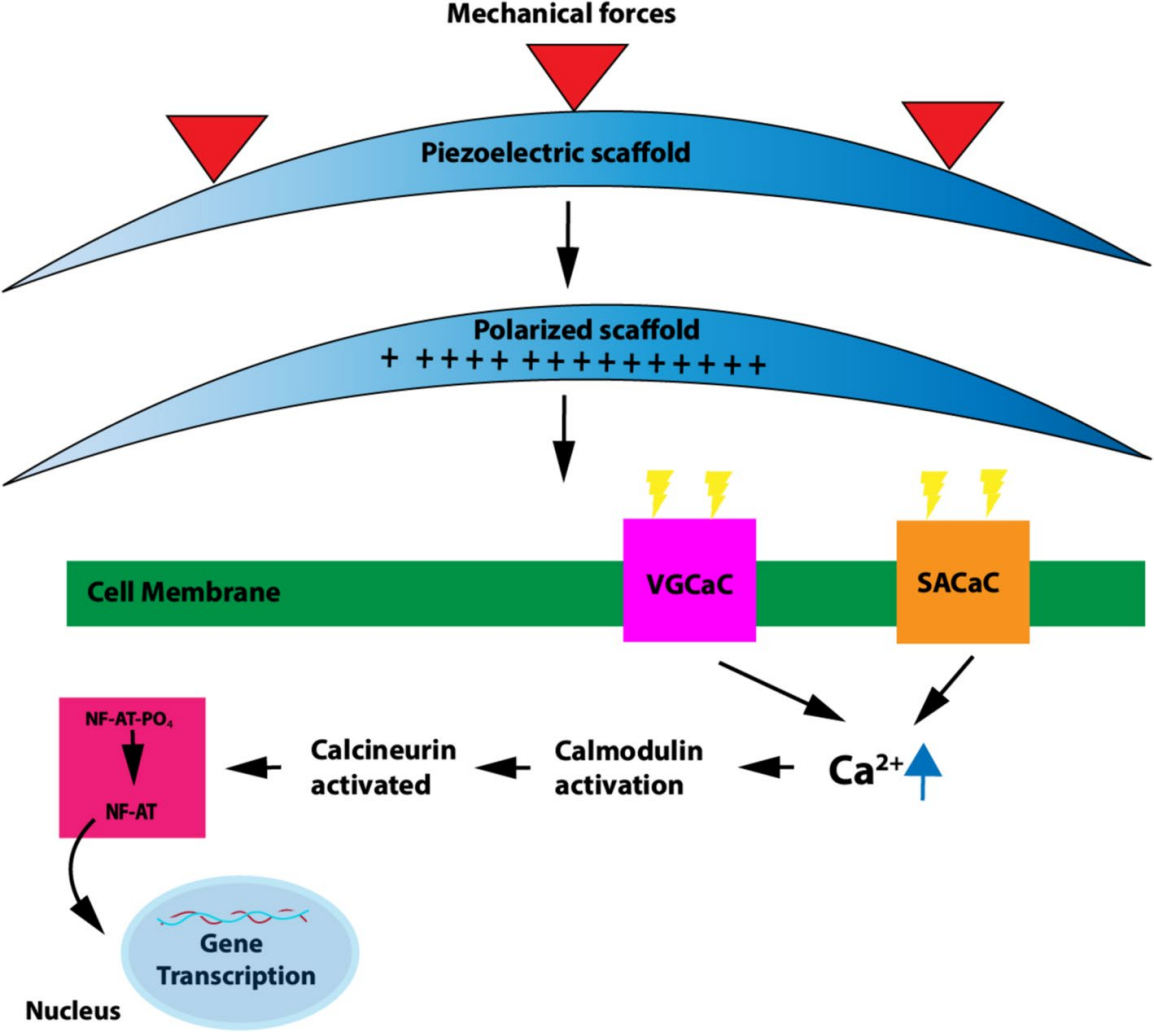
Potential cellular response to piezoelectric stimulation. Mechanical forces imposed on the piezoelectric scaffold induce deformation. The deformation polarizes the charge of the scaffold. Once polarized, electrical signals are generated and released onto the cell membrane. The electrical response is directed towards the voltage-gated and stretch-activated calcium channels, respectively. The polarization of the channels forces an opening in which calcium ions are released into the cell, increasing Ca^2+^ concentration (Leppik et al. 2020). The influx of calcium activates calmodulin (calcium-binding protein). Calmodulin activation along with increase in Ca^2+^ concentration is responsible for further activation of calmodulin-dependent serine/threonine protein phosphatase known as calcineurin. NF-AT, a family of factors responsible for gene transcription is dephosphorylated by calcineurin and translocated to the nucleus (Jacob et al. 2018). When new DNA is transcribed, the cells within the gene gain the capability of differentiation

Piezoelectricity is an attribute of material asymmetry, which leads to the spontaneous generation of electric signals upon mechanical deformation (Jeon et al. 2020; Ning et al. 2018). Electroactive materials that possess electric and electromechanical clues have been proven to be highly relevant in changing cellular behavior and promoting regeneration of a variety of tissues such as bone, cartilage, skin and muscle (Jeon et al. 2020; Liu et al. 2022c) (Table 2). Standard in vivo electric stimulation requires implanted power sources, however it has many drawbacks including greater invasiveness in surgery, a risk of infection, potential of lead breakage from implanted battery due to impact, as well as tissue irritation and damage due to incompatible mechanical properties at the electrode-tissue interface (Wen and Liu 2014). Piezoelectric materials have the capability to deliver electric stimulation to tissues without a need for external power sources (D'Alessandro et al. 2021; Leppik et al. 2020) (Fig. 2). Piezoelectric stimulation distinguishes itself from active electrical stimulation because electrical signals are not directly delivered from a power source, but rather produced as a result of mechanical force. The ability for piezoelectric materials to convert mechanical forces into electrical signals are due to the inherent material characteristics. One potential mechanism responsible for piezoelectric-induced cell differentiation is through electrical signals induced by the mechanical stimulation, which open calcium channels and cause an increase in Ca^2+^ concentration, affecting transcriptional changes reflected in cellular response (Jacob et al. 2018; Leppik et al. 2020; Liu et al. 2022a) (Fig. 3). Piezoelectricity is also naturally present within the body and plays an important role in bone physiology due to a high concentration of collagen (Ning et al. 2018). Studies have focused on studying the therapeutic potential of piezoelectric materials on the human body, including nerve, bone, skeletal muscle, cartilage, heart, and others.

**Table 2:** Applications of piezoelectric stimulation

Application	Material	Results	Ref
Neural	PVDF-TrFE	The in vitro 3D neuron-glial interface was induced by mechanoelectrical stimulation, which resulted in enhanced interactions among cellular complements and improved neural connectivity and function. Differentiation toward neurons, oligodendrocytes, and astrocytes were observed following piezoelectric stimulation	Tai et al. 2021
Neural	PVDF-TrFE	The Nut-PNPs showed decreased viability of the cells in vitro with respect to controls	Pucci et al. 2022
Neural	AF and NP samples (annulus fibrosus & nucleus pulposus)	Longitudinal piezoelectricity on in vitro samples can induce voltages of 0.38 to 1.5 nV locally through IVD which can affect cell alignment	Poillot et al. 2020
Neural	PVDF/PCL hybrid	Following in vivo implantation on nervous tissue, 9.1% of PVDF/PCL scaffolds were degraded after 4 months	Cheng et al. 2020
Bone	PVDF-PPy	PVDF-PPy promoted in vitro MSC osteogenic differentiation	Zhou et al. 2019
Bone	PVDF-CFO	in vitro MSC culture was viable on PVDF-CFO supports with increased proliferation	Guillot-Ferriols et al. 2020
Bone	PVDF	Human MSCs proliferated and exhibited in vitro osteogenic differentiation on electrosprayed PVDF	Sobreiro-Almeida et al. 2017
Bone	BaTiO_3_ upon Ti6Al4V	Bone formation was observed in the in vivo spinal model with increasing implantation time	Liu et al. 2020a, b
Cartilage	PHBV	Poled in vitro samples and those with the electrical field applied have shown to have greater chondrocyte proliferation and cell activity	Jacob et al. 2019
Skeletal muscle	PVDF	With or without surface charge, PVDF film supports in vitro myogenic differentiation. Charged surfaces had higher maturation and fusion indexes than the controls showing that electric stimulation improves differentiation of muscle cells into myotubes	Ribeiro et al. 2018

### Piezoelectric materials

#### PVDF

Polyvinylidene fluoride (PVDF) has been frequently used in tissue engineering given excellent non-toxic properties, biocompatibility, flexibility, and piezoelectric functionality (Ahmadi et al. 2019). It has a semi-crystalline structure with one of the highest known piezoelectric coefficients, which results from the strong dipole moment generated by the electronegativity difference between fluorine and hydrogen atoms (Martins et al. 2014). Due to its hydrophilicity and inert nature, PVDF can be chemically modified and mixed with other polymers to maximize its piezoelectric properties (Qazi et al. 2015). For example, Polyvinylidene fluoride-hexafluoropropylene (PVDF-HFP) is a derivative of PVDF that possesses piezoelectricity and biocompatibility. A major difference that distinguishes it from its predecessor is its bioinert nature making it ideal for the purpose of providing structure in anatomy. It is widely studied in cardiovascular systems as a material for coronary stents (Baumgartner et al. 2022). Polyvinylidene fluoride-trifluoroethylene (PVDF-TrFE) is another common derivative of PVDF that shares its qualities of piezoelectricity and biocompatibility, but it exhibits greater remnant polarizations, which yield larger electrochemical coupling factors (Jia et al. 2017). As a result, it has greater efficiency in transforming from mechanical cues to electrical.

#### PZT

Lead zirconate titanate (PZT) has been shown to produce a current of > 1 mA in response to an applied 1 MHz ultrasound transmitted into the body (Wen and Liu 2014). It has high sensitivity to ultrasound frequency > 35 kHz allowing its outputs to be easily evaluated (Li et al. 2015). PZT has a Curie temperature of 300 °C and can operate at a temperature of up to 200 °C.

#### BNNT

Boron nitride nanotubes (BNNT) have a strong piezoelectricity and established biocompatibility as demonstrated in previous studies (Ricotti et al. 2013). A drawback of BNNT is its tendency to form large clusters in aqueous media, which hinders its direct use for cell studies. Strategies have been formulated with a wide range of polymers such as glycol chitosan (GC), poly-L-lysine and polyethyleneimine to allow uniform dispersion (Ricotti et al. 2013).

#### KNN

Potassium-sodium niobate (KNN) is a thin, ceramic film based on potassium sodium niobate (Gaukas et al. 2020). It has a high Curie temperature and maintains good piezoelectric properties. KNN is a lead-free substrate with low acute toxicity. There are numerous environmental concerns associated with lead-based piezoceramics. Lead-free films have been increasing extensively researched over the past two decades, signifying that KNN is growing in use.

#### PLLA

Poly(L-lactic acid) is a biodegradable, nontoxic piezopolymer. Piezopolymers present attractive properties for therapy as they are mechanically flexible and biocompatible (Liu et al. 2022c). PLLA has been shown to exhibit high polarization levels due to its α-, β-, and γ-crystalline forms. The β-crystalline form has been shown to exhibit the highest polarization of the three and has excellent piezoelectric properties.

#### PHB

Polyhydroxybutyrate (PHB) has shown excellent biodegradable and biocompatible properties in a variety of different tissue types for in vivo and in vitro use (Castellano et al. 2018). It has exhibited biocompatibility with wound dressing, cardiac repair, bone, and nerve tissue (Timin et al. 2018). Compared to other polymers with large piezoelectric constants like PVDF, PHB has insufficient piezoelectric charge constant values (Timin et al. 2018). PHB’s piezoelectric properties can be improved when applied in conjunction with other conductive polymers (Qazi et al. 2014).

#### PHBV

Poly(3-hydroxybutyrate-co-3-hydroxyvalerate) (PHBV) is a good candidate for biomedical uses due to its high biodegradability and biocompatibility. It has a higher degradation time than most other biocompatible polymers, enabling it to maintain mechanical integrity in hard tissue applications (Jacob et al. 2019). In addition, it offers high absorption capacity, low cytotoxicity, and thermoplasticity in addition to piezoelectric properties (Jacob et al. 2019). It has significant applications in tissue engineering, including implants, tissue patches, and scaffolding, making it applicable for medical use like cardiovascular stents, drug delivery, and wound enclosures.

#### nAK

Akermanite (nAK) is a calcium silicate with a high piezoelectric coefficient. It has gained attention due to its status as a lead-free piezoelectric bioceramic. It has controllable mechanical properties and degradation rate (Shokrollahi et al. 2017). Human bone marrow-derived stromal cells exhibited proliferation and differentiation when acted upon by akermanite scaffolding (Sun et al. 2006).

### Neural applications

The effects of piezoelectricity have been investigated for a variety of neural applications. Neonatal rat and adult human Schwann cells exhibited minimal toxicity on PVDF and PVDF-TrFE scaffolds (Gryshkov et al. 2021). Dorsal root ganglions (DRGs) displayed long neurite extension in all directions with great uniformity cultured on PVDF-TrFE scaffolds. A similar study reported that PVDF-TrFE scaffolds supported Schwann cell growth and neurite extension (Wu et al. 2018). Schwann cells showed myelinating characteristics with positive staining for myelin basic protein (MBP) and Caspr (paraanodal domains of a myelin sheath) in DRG co-cultured on PVDF-TrFE (Wu et al. 2018). PVDF-TrFE was electrospun into nano and micro-sized fibers under annealing to further enhance mechanical and piezoelectric properties. Neurite extension was greatest on aligned, annealed PVDF-TrFE having micron-sized fiber dimensions compared to limited radial extension observed on random scaffolds (Lee et al. 2011). Use of externally applied oscillating electrical fields via polarized PVDF scaffolds further increased neuronal density 115% and neurite count by 79% using a mixed rat spinal cell culture, respectively (Royo-Gascon et al. 2013). The PVDF/PCL hybrid scaffolds with improved mechanical strength and biocompatibility were implanted in the transected rat sciatic nerve model to promote peripheral nerve regeneration. The PVDF/PCL scaffolds showed positive effects on the myelination and axon regeneration, indicated by markers such as MBP, β3-tubulin, S100 and neurofilament protein 160 (NF 160) (Cheng et al. 2020). The regenerated nerves exhibited comparable results to autografts regarding electrophysiological, morphological and functional restoration after 4 months of implantation.

Lead-free piezoelectric ceramics KNN was used to investigate biocompatibility, in which rat Schwann cells and human fibroblast cells showed higher cell proliferation rates than glass samples due to piezoelectric effects (Gaukas et al. 2020). Doping KNN thin films with CaTiO3 demonstrated similar biological effects (e.g. proliferation rate, cell morphology, viability) compared to KNN thin films and platinized silicon. PVDF-based piezoelectric composites such as incorporating PVDF/MCM41 (mesoporous silica nanoparticles) into gellan/polyaniline/graphene scaffolds were developed to evaluate PC12 proliferation (Mohseni et al. 2021). This study showed increased cell proliferation over 3 days without cellular toxicity using the piezoelectric nanocomposite. Piezoelectric ceramic lead zirconate titanate (PZT) increased axonal length of rat cortical neuron cells, however it significantly decreased cell density (Wen and Liu 2014). It is unclear if lead PZT induced some level of toxicity in these cells. Authors concluded changed electrophysiological characteristics following PZT culture such as increased amplitude and frequency of excitatory postsynaptic (EPSC) currents, which they contributed to downregulated Netrin-1 and its receptor Deleted in Colorectal Cancer (DCC) via the Rho GTPase signaling pathway (Wen and Liu 2014).

Piezoelectricity induces differentiation of neural stem cells. Using electrospun PVDF-TrFE nanofibers as a cell culture scaffold, authors created a three-dimensional neuron-glial interface in vitro with improved neural connectivity and functionality (Tai et al. 2021). They examined the effects of electrical stimulation, mechanical stimulation (e.g. hydro-acoustic actuation), or mechano-electrical stimulation on the differentiation capacity of neural stem cells toward neuronal, oligodendrocytic, or astrocytic lineages. All conditions showed an increase of neuronal markers (e.g. Tubb3). However, electrical stimulation in the presence of mechanical stimulation promoted neural stem cell differentiation and maturation toward myelinating oligodendrocytes. Mechanical stimulation and mechano-electrical stimulation conditions also enhanced expression of astrocytic genes. Greater connectivity of extracellular neuronal activities was observed by multielectrode array in the mechano-electrically stimulated constructs as compared to those in the biochemically mediated condition or statically scaffold-cultured condition (Tai et al. 2021). The mechano-electrical concept was also further developed for anticancer treatment. A nanoplatform consisted of nutlin-3a-loaded ApoE-functionalized PVDF-TrFE nanoparticles was remotely activated with ultrasound-based mechanical stimulations to induce drug release and to locally deliver anticancer electric cues (Pucci et al. 2022). Nutlin-loaded PVDF-TrFE nanoparticles exerted only a mild cytotoxic effect on T98G glioblastoma cells, however the induction of ultrasound significantly improved their therapeutic efficacy by reducing cell migration, actin polymerization, and invasion ability due to potential pathways related to cell division, autophagy, and cell adhesion. The piezoelectric property could also result from the non-centrosymmetric molecular structure in chitosan (Silva et al. 2001). Following deformation of mechanical samples that included CS fibers coated with PEDOT and PEDOT: PSS respectively, those listed nanofibers exhibited greater neurite length and cell proliferation rate (Du et al. 2020). The longitudinal piezoelectricity showed voltages of 0.38 to 1.5 nV locally through intervertebral discs (IVDs) from dissected bovine tails (Poillot et al. 2020). The organized collagen networks in the annulus fibrosus created a greater piezo response than the nucleus pulposus.

### Musculoskeletal applications

Piezoelectric stimulation is commonly used in orthopedic research. Previous study consisted of dynamic piezoelectric stimulation on an electroactive polypyrrole-coated polyvinylidene fluoride (PVDF-PPy) composite showed an active osteogenic promotion from mesenchymal stem cell (MSC) differentiation (Zhou et al. 2019). In addition, MSC adhesion, spreading, and elongation were all favored by the PVDF-PPy composite in comparison to a base PVDF scaffold (Zhou et al. 2019). This evidence suggested that piezoelectric stimulation was capable of biasing MSCs toward osteogenic fate. The electro-spraying of a PVDF film (film is coated with a liquid emitted with high voltage) also exhibited osteogenic differentiation in MSCs following the stimulation (Sobreiro-Almeida et al.^ 2017^). The differentiation of rat MSCs into osteogenic and chondrogenic lineages was observed with high and low piezoelectricity values on the polarized PLLA (poly-lactic acid) under electrical field, respectively (Liu et al. 2022c). Specifically, after mechanical deformation, osteogenic differentiation occurred with an increased modulation of calcium-binding proteins and intracellular transients (Liu et al. 2022c). Modifications to the mechanical property of PVDF films also affect piezoelectric potential. The PVDF was first modified with polyvinyl alcohol (PVA) to increase hydrophilicity and improve biocompatibility (Ahmadi et al. 2019). The Ba0.9Ca0.1TiO3 powder was added to enhance tensile strength of the modified fibrous membrane. The addition of Ba0.9Ca0.1TiO3 not only augmented piezoelectric potential with increased voltage signals but also significantly increased MSC proliferation (Ahmadi et al. 2019). This demonstrates the importance of tuning mechanical properties of the underlying substrate to maximize piezoelectric induced biological response.

PVDF substrates containing cobalt ferrite oxide (CFO) showed increased proliferation of MSC compared to unmodified PVDF (Guillot-Ferriols et al. 2020). Investigations into the piezoelectric potential of scaffolds devoid of PVDF have expanded the range of possible osteogenic differentiation and regeneration treatments. To test the biocompatibility of the piezoelectric bio-ceramic made of barium titanate and nano-akermanite composite, in vitro results showed an 85% MSC survival in viability following 7 days of culture on the scaffold (Ricotti et al. 2013). It is crucial to maintain cell survival in addition to a high piezoelectric coefficient for enhanced biological response. A sample with the highest proportion of akermanite specifically was found to be the most non-toxic, making akermanite an essential element for supporting cell viability. Other non-PVDF materials include piezoelectric scaffolds containing substances such as PHB, polyhydroxybutyrate-polyaniline (PHB-PANI), and polycaprolactone (PCL). Bone marrow-derived human MSCs cultured on PHB and its derivative PHB-PANI exhibited significantly more cell viability, adhesion, and proliferation (~ 40%) than PCL (Timin et al. 2018). MSCs cultured on PHB and PHB-PANI in osteogenic medium further showed matrix mineralization, suggesting these materials are potentially suitable for bone treatment. A study investigated the implantation of a titanium alloy substrate scaffold coated with piezoelectric nanoparticles (BaTiO3) into a sheep’s spine. X-rays showed that the piezoelectric effect of the scaffold reinforced osteogenesis and bone formation increased with implantation time (Liu et al. 2020b). A hybrid piezoelectric material made of poly(3-hydroxybutyrate-co-3-hydroxyvalerate) (PHBV) and barium titanate BaTiO3 was used to evaluate its interactions with MSC differentiated chondrocytes (Jacob et al. 2019). Polarized hybrid scaffolds promoted chondrocyte attachment, proliferation, and collagen II expression. Collagen II is a major structural component of articular cartilage in the extracellular matrix. The polarized hybrid scaffolds could potentially be used for cartilage tissue applications. Bone itself has been shown in the past to have piezoelectric capabilities due to its structure of collagen which has been shown to create electric signals in response to mechanical loads (D'Alessandro et al. 2021). Based on this concept, a study was conducted with the purpose of mimicking the spatially specific piezoelectricity within bones. A material made of two parallel interspersed domains with differing piezoelectric coefficients were laser irradiated to produce microscale piezoelectric zones (MPZ) (Yu et al. 2017). An animal implantation component was present in this experiment as MPZ samples were placed into New Zealand rabbit femoral condyles. Bone growth was observed 4 weeks after implantation, exhibiting the scaffold’s ability to induce bone regeneration without the need for exogenous cell sources (Yu et al. 2017).

Electrical stimulation not only influences muscle cell phenotype, myosin expression and contractile sarcomere assembly, but also modulates fiber type switch and induces contractility in differentiated myotubes (Qazi et al. 2015). C2C12 myoblasts, a murine myoblast cell line, is routinely used as an experimental model of skeletal muscle. To understand the regeneration potential of skeletal muscle through piezoelectric polymers, C2C12 cells were cultured on β-PVDF films exhibiting different types of charged surfaces to quantify the differentiation capabilities such as the fusion and maturation index (Ribeiro et al. 2018). Myogenic differentiation was supported by the PVDF film. Charged surfaces improved the fusion of muscle cells into differentiated myotubes, as demonstrated by fusion and maturation index values higher than the neutral-charged controls (Ribeiro et al. 2018). Similarly, researchers conducted in vitro experiment measuring the myogenic differentiation of C2C12 with internalized piezoelectric boron nitride nanotubes (BNNTs) (Ricotti et al. 2013). An ultrasound was applied daily after BNNTs were added to cell culture and internalized by cells. BNNT-mediated stimulation enhanced expression of Myogenin, Muscle LIM Protein (MLP), and MHC-IIa (MYH2) markers which indicate myogenesis on C2C12 cells. BNNT samples were also characterized with a higher proportion of elongated multinucleated myotubes (Ricotti et al. 2013). However, co-cultured fibroblasts showed no change in differentiation ability in BNNT mediated conditions as no remarkable difference was observed in expression of extracellular matrix (ECM) proteins (Ricotti et al. 2013). Authors reasoned that BNNTs were internalized only by the C2C12 cells on the top layers, while no particles were internalized by the underneath fibroblast layer (Ricotti et al. 2013). This study highlights that piezoelectric induced biological response can vary in its effectiveness due to the route of delivery/stimulation and different cell and tissue types despite the same stimulation platform.

## Magnetic-field induced stimulation

**Fig. 4:**
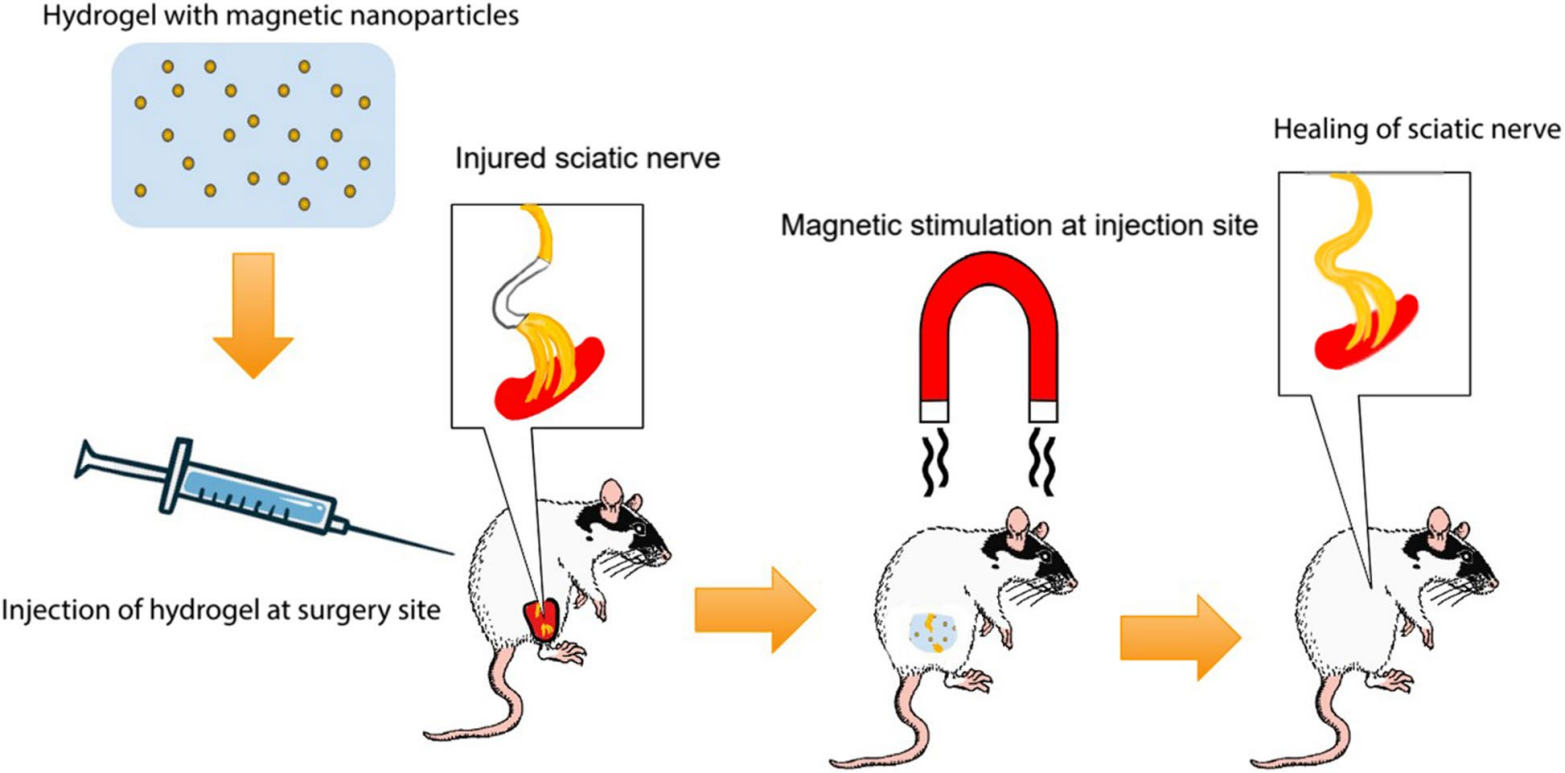
The use of magnetic hydrogel as a minimally invasive treatment for sciatic nerve repair. A magnetic hydrogel is synthesized by uniformly dispersing magnetic nanoparticles through a hydrogel. The magnetic hydrogel is then injected into a rat with a damaged sciatic nerve. The magnetic hydrogel is stimulated with an external magnetic field. The magnetic stimulation induces directionally oriented axonal growth, leading to sciatic nerve regeneration and recovery. The sciatic nerve is healed with natural degradation of magnetic hydrogel within the body

Magnetic stimulation as a minimally invasive treatment is employed through the use of magnetic nanoparticles. Magnetic nanoparticles made of gold and iron oxide are dispersed in forms of mediums such as hydrogels and scaffolds through crosslinking or in situ formation (Adams et al. 2016; Sirkkunan et al. 2021). Once magnetic hydrogels are applied to the site of injury, encapsulated nanoparticles can be magnetically guided and activated via external magnetic stimulation to promote biological response (Manas-Torres et al. 2021) (Fig. 4). The mediums used for magnetic stimulation have several key properties that are critical to external stimulation for desired biological responses. Among them are biocompatibility, biodegradability, and the ability to encapsulate the magnetic nanoparticles. More specialized properties including cellular adhesion and swelling require specific material synthesis (Huang et al. 2019; Omidinia-Anarkoli et al. 2017). These magnetic hydrogels have paved a new era in minimally invasive therapy and have made it feasible to treat deep internal sites without the need of multiple surgeries. Potential applications of magnetic stimulation include neural regeneration, osteogenic repair, hyperthermia treatment, and drug and gene delivery (Table 3).

**Table 3:** Applications of magnetic stimulation

Application	Material	Magnetic Nanoparticles	Results	Ref
Neural	Collagen (type-I, rat tail extract) hydrogel	magnetic nanoparticle-decorated reduced graphene oxide (GO, Fe_3_O_4_)	Hydrogel-encapsulating SH-SY5Y showed cell differentiation and extensive neurite growth	Santhosh et al. 2019
Neural	Plain collagen scaffolds, collagen Mimetic Peptides	Gold magnetic nanoparticles	Magnetic field treatment significantly increased PC12 neurite length and alignment	Sirkkunan et al. 2021
Neural	Pre-formed collagen hydrogels	Fe_3_O_4_	Magnetic field safely enhanced MNP-mediated gene delivery to NSCs grown on collagen gels	Adam et al. 2016
Neural	PLLA	Fe_3_O_4_	Magnetically responsive aligned poly-l-lactic acid electrospun scaffolds increased DRG neurite length and alignment	Johnson et al. 2019
Neural	magneto-responsive PLGA	Fe_3_O_4_	The in vitro study showed unidirectional DRG growth along the fiber orientation	Omidinia-Anarkoli et al. 2017
Neural	Alginate-magnetic short nanofibers 3D composite hydrogels	Fe_3_O_4_	The hydrogel induced differentiation of OE-MSCs into neuron- and glial-like cells	Karimi et al. 2021
Neural	Silk Fibroin/Gelatin (SG) Hydrogels	Fe_3_O_4_-graphene	Electrical pulses significantly increased PC12 neurite elongation	Lin et al. 2020
Neural	S.platensis, BaTiO_3_ nanoparticles	Fe_3_O_4_	PC12 showed increased neurite outgrowth length using the micromotor under ultrasound exposure within 3 days	Liu et al. 2020a, b
Neural	S.platensis, BaTiO_3_ nanoparticles	Fe_3_O_4_	In vitro differentiation of astrocytes, neurons, and oligodendrocytes could be controlled with ultrasound frequencies	Liu et al. 2021a, b
Neural	amphiphilic polysaccharide nanogels containing cholesterol-bearing pullulan	Fe_3_O_4_	Differentiation of ADSCs to neuron-like cells occurred within a week by magnetic induction of exosomes	Mizuta et al. 2019
Neural		Fe_3_O_4_	Higher rate of neurosphere formation was observed under magnetic field	Li et al. 2021
Neural	Nerve growth factor	Fe_3_O_4_	PC12 demonstrated neurite outgrowth. The in vivo study showed that magnetic nanoparticles could be localized along the magnetic path in tissues without adverse effects	Marcus et al. 2018
Neural	hyaluronic acid/collagen (HA/Col), TiO_2_, tetrabutyl titanate	Fe_3_O_4_@BaTiO_3_	After 30 days of implantation, axonal regeneration was observed under magnetoelectric treatment in rats with hemi-transected spinal cord injury	Zhang et al. 2021
Neural	glycidyl methacrylate- HA hydrogel	Fe_3_O_4_	Regenerated axons were detected in rats with transected sciatic nerves after 4 weeks	Lacko et al. 2020
Neural	gelatin-genipin hydrogel	Fe_3_O_4_	Magnetic stimulation demonstrated improved locomotor recovery in rat contusive spinal cord injury by increasing level of neurotrophins, decreasing inhibitory molecule concentration, and reducing microglia activity and glial scarring	Bhattacharyya et al. 2021
Bone	type II collagen, HA, and polyethylene glycol (PEG) hybrid hydrogel	Fe_3_O_4_	Magnetic nanoparticles responded to external magnetic stimulation and cell adhesion was not negatively affected	Zhang et al. 2015
Cartilage	hyaluronic acid–polyacrylic acid hydrogel	Fe_3_O_4_	The hydrogel provided a protective environment for chondrocyte migration and enhanced the recovery of damaged cartilage to achieve a new smooth surface in rabbits after 8 weeks	Chiang et al. 2021
Cartilage	Gelatin, β-cyclodextrin hydrogel	Fe_3_O_4_	More than 80% of the cartilage defects were filled at 8 weeks, and defects were completely repaired by 12 weeks	Huang et al. 2019
Skeletal muscle	Chlorella pyrenoidosa	Fe_3_O_4_	The CP@Fe_3_O_4_ microswimmer induced significant contraction of targeted C2C12 myotubes as well as in exposed muscles	Liu et al. 2022a, b

### Neural applications

One advantage of magnetic hydrogels in neural regeneration is the ability of magnetic stimulation to enhance cellular properties. Neural stem cells (NSCs) were grown on the pre-formed collagen hydrogels and added with magnetic iron oxide (Fe_3_O_4_) nanoparticles to investigate the magnetiofection (Adams et al. 2016). NSC stimulated under an oscillating magnetic field displayed enhanced green fluorescent protein levels through this nanoparticle mediated gene delivery. This demonstrates that magnetofection is a potential methodology to genetically engineer stem cells for neural applications. The effects of magnetic stimulation of NSCs were also examined using superparamagnetic iron oxide nanoparticles (SPIONs) (Li et al. 2021). The SPIONs were incubated with the NSCs to determine the uptake of SPIONs into the cell. In the absence of the static magnetic field, NSCs with greater concentrations of SPIONS exhibited more neurospheres, indicating increased cell proliferation. When exposed to a static magnetic field of 50 ± 10 mT, NSCs with high concentrations of SPIONs resulted in fewer neurospheres despite maintaining similar sphere diameters.

Magnetic stimulation has been shown to encourage cell differentiation and guide directional axonal growth. A study found that human neuroblastoma SH-SY5Y cells cultured on synthesized collagen hydrogels containing iron oxide and magnetic graphene oxide nanoparticles not only promoted neurite growth, but also caused cells to be directionally oriented (Santhosh et al. 2019). While the collagen hydrogel was solidifying, a low magnetic field (~ 50 mT) was applied by magnetically aligning the field responsive graphene oxide (m-rGO) nanoparticles to orient directional gelation of the collagen fibers. The resulting neurites were found to have lengths of 181.05 ± 35.8 µm and had growth in the direction of the magnetically aligned collagen fibers. Similarly, using collagen hydrogel with gold magnetic nanoparticles induced directional axonal growth in neurites (Sirkkunan et al. 2021). While the injectable hydrogel was tested on PC12 cells as opposed to SH-SY5Y cells, the magnetically templated scaffold led to neurite growth and alignment. Specifically, gold magnetic nanoparticles (GMNP) were combined with collagen mimetic peptides and stimulated with a low external magnetic field to align the collagen fibers. This collagen scaffold was synthesized to have remote control of the fiber alignment within injectable collagen as most injectable materials lack defined microarchitecture that is normally found from in vitro patterned scaffolds (Sirkkunan et al. 2021). The injectable GMNP/collagen hydrogels showed excellent PC12 cell alignment with 86% of the neurites displaying alignment within 30 degrees of the magnetic field and a length of 67.48 μm. The difference in neurite length in these two studies could be due to the cell type as well as the concentration of magnetic nanoparticles in use. The concentration of magnetic nanoparticles in the scaffold is responsible for the magnetic responsiveness of the hydrogel and alignment of collagen fibers, which contribute to the directional neurite growth. Using a lower concentration of magnetic nanoparticles may have led to decreased sensitivity to external stimulation, resulting in less directional growth and shorter neurites.

Magnetic nanoparticles are used for microarchitecture formation inside scaffolds to enable cellular adhesion after magnetic nanoparticles are removed. Magnetic alginate microparticles (MAMs) formed aligned structures within a glycidyl methacrylate hyaluronic acid and collagen I hydrogel scaffold with tubular microarchitecture to support cellular adhesion and remodeling (Lacko et al. 2020). The structured scaffold was cultured with DRGs to test biocompatibility, biodegradability, and cell behavior. Over 7 days, DRG axons were extended and penetrated the structured hydrogel. Hydrogel was implanted into rats with transected sciatic nerves for 4 weeks. Regenerated axons were observed over the 10 mm defect with increased fiber density. A previous study created a gelatin-genipin hydrogel with iron oxide nanoparticles (IONPs) and exposed the rats to magnetic stimulation to treat rat contusive spinal cord injury (Bhattacharyya et al. 2021). Animals were exposed to a uniform magnetic field for 2 h/day for 5 weeks. A significant locomotor improvement was observed based on Basso, Beattie, and Bresnahan scores along with enhanced GAP-43 expression, indicating potential nerve repair. The magnetic field and IONPs altered the microenvironment to be conducive to neural repair and regeneration. The combination of ECM mimicking scaffolds and magnetic stimulation has potential to be effective for promoting neural recovery in animals (Bhattacharyya et al. 2021; Lacko et al. 2020).

Magnetic nanoparticles can act as a delivery system to target specific cells and locations by guiding nanoparticles with an external magnetic field. Previous study isolated exosomes from PC12 cells and combined them with a magnetic nanogel to deliver exosomes efficiently into targeted cells to induce differentiation (Mizuta et al. 2019). The magnetic nanogel was formed using oleic acid coated Fe_3_O_4_ nanoparticles combined with a cholesteryl-group-substituted pullulan (CHP) nanogel. A magnet with a magnetic flux density of 0.5 T was applied to the hybrid nanogel and guided to the targeted adipose-derived mesenchymal stem cells (ADSCs). The neuronal differentiation of ADSCs was observed within 7 days and neurite outgrowth was found to be in the direction of applied magnetic field. One could combine nerve growth factor (NGF) with magnetic nanoparticles (MNPs) to allow site specific NGF delivery with an external magnetic field (Marcus et al. 2018). Using seven neodymium magnetic rods arranged in a circle with one in the middle, a field of 0.16 T was applied to the NGF-MNP complex to guide it to certain areas within the culture dish. The PC12 cells within the dish were incubated for 4 days to observe local differentiation. Near the magnetic rods, there was significant neurite growth with interconnected matrix with their neighbors. Cells further from the magnetic sites showed either shorter neurites or no differentiation at all. NGF-MNP complex was injected into mouse sciatic nerves and guided with an external magnet before nerve tissues were harvested. A clear accumulation and localization of the MNPs along the magnet’s path was observed, indicating that the complex could be guided to specific sites. Furthermore, no negative effects of NGF-MNP complex were observed over 18 days in mice, indicating that the complex could be safely used in animals to facilitate localized molecule delivery for promoting neural regeneration.

Magnetically oriented nanofibers provide a stable structure for the growth of neurites and are highly effective at inducing directional growth (Johnson et al. 2019; Omidinia-Anarkoli et al. 2017), which is vital for mimicking the natural extracellular matrix of repair sites. Comparing to using pre-formed topography to guide cellular behavior (Lin et al. 2020), magnetically oriented patterns can be formed only upon magnetic field exposure, controlling precise scaffold architectures for neurite extension. Additionally, the presence of magnetic nanoparticles within the oriented nanofibers would increase cell proliferation and growth (Johnson et al. 2019; Karimi et al. 2021; Omidinia-Anarkoli et al. 2017), which could further stimulate nerve repair. Researchers used magneto-responsive poly (lactide-co-glycolide) (PLGA) fibers within an anisotropic hybrid hydrogel (Anisogel) to induce unidirectional cell growth (Omidinia-Anarkoli et al. 2017). The PLGA fibers were combined with SPIONS and oriented within a biocompatible fibrin hydrogel to form the Anisogel. The Anisogel was cultured with dorsal root ganglia (DRGs) for 7 days to study the orientation of the neurites within the gel and the cytotoxicity of the Anisogel. The Anisogel led to a 55% increase in neurite length and a highly directional formation. Neurites grew parallel to PLGA fibers with a full width half maximum (FWHM) of 59.6° ± 15.4°, which was narrower than the controls with randomly oriented fibers and no fibers. Another research group used a similar method in which electrospun poly-l-lactic acid (PLLA) fibers were integrated with 6% weight SPIONs to increase neurite growth (Johnson et al. 2019). The magnetically responsive fibers were injected into a collagen or fibrin hydrogel and then magnetically oriented using an external magnetic field. The biocompatibility and neurite orientation were tested using DRGs within a collagen/Matrigel hydrogel and a fibrin/Matrigel hydrogel. The mean neurite length increased 1.4 and 3 times for collagen/Matrigel and fibrin/Matrigel hydrogels, respectively. Neurites in contact with the fibers in both hydrogels grew and aligned along with fibers, whereas non-contact neurites formed radial patterns with no clear orientation. Alginate-magnetic short electrospun nanofibers were also used to encapsulate olfactory ecto-mesenchymal stem cells (OE-MSCs) for potential neuronal regeneration (Karimi et al. 2021). Magnetic fibers were formed by loading SPIONs into wet-electrospun gelatin nanocomposite fibers, which were then embedded in an alginate hydrogel. This formed a magnetic short nanofiber (M.SNF)/hydrogel. A significant increase of OE-MSC proliferation was found within the M.SNF/hydrogel, confirming hydrogel biocompatibility. The M.SNF/hydrogel also induced differentiation of OE-MSCs indicated by increased neuronal marker β-tubulin III and glial marker GFAP. Cells exposed to SPIONs were more likely to differentiate toward neuronal lineage, which could be important for nerve regeneration and repair. A silk fibroin/gelatin (SG) hydrogel was synthesized with corrugated patterns on the surface to maximize surface interactions for cell adhesion and oriented neurite growth (Lin et al. 2020). Reduced graphene oxide (rGO) was combined with Fe_3_O_4_ and nerve growth factor (NGF) to form nerve growth factor-incorporated Fe_3_O_4_−graphene nanoparticles (GFPNs), which were magnetically deposited within the hydrogel. The corrugated SG with GFPN patterns was cultured with PC12 cells under electrical stimulation, in which an electric pulse (EP) of 250 μA and the duration of 5 ms were applied in two rounds of 5 min of stimulation with a 3-min break. After 14 days, neurite alignment on the corrugated SG was within 15° for 10 μm GFPN pattern, but alignment decreased as pattern dimension increased. Cell density increased with greater pattern dimension due to the increased surface area for cellular adhesion. Specifically, the SG with corrugation patterns of 30 μm resulted in optimal cell adhesion and differentiation in response to the pattern guidance. The addition of EP on GFPN-deposited SG showed a1.5-fold increase in the neurite elongation as early as 7 days. The guidance and increase in neurite length allow for the potential development of nerve conduits.

Magnetic stimulation can be uniquely applied in combination with other wireless stimulation methods. A previous study coated *S. platensis* with magnetic Fe_3_O_4_ nanoparticles and piezoelectric BaTiO_3_ nanoparticles to create a wireless controllable micromotor (Liu et al. 2020a). By applying a low-intensity rotating magnetic field (e.g. f ≈50 G with a rotational speed of 120 rad min − 1), the micromotor was steered towards a single PC12 cell. The micromotor was then exposed to an ultrasonic field, through which piezoelectric nanoparticles could generate electrical stimulus for in situ cell differentiation. Specifically, PC12 showed neurite outgrowth using the micromotor under ultrasound exposure within 3 days. After 5 days, very few traces of the micromotor could be observed, indicating its short-term biodegradability. The authors applied the wireless controlled micromotor to rat neural stem cells for 4 days (Liu et al. 2021a). They found that the ultrasound frequency determined the cell differentiation. With an ultrasound input of 0.5–0.9 W/cm^2^, most cells differentiated into astrocytes. Dopamine and cholinergic neurons were likely to form at 1.0—1.5 W/cm^2^ and 1.5–2.0 W/cm^2^, respectively. A 2.1–2.5 W/cm^2^ input is preferred by oligodendrocyte differentiation. This minimally invasive and highly specific targeting system has great advantages to individually target and differentiate single cells. It is possible to combine piezoelectric materials, magnetic nanoparticles, and a hyaluronan/collagen to form a Fe_3_O_4_@BaTiO_3_ NPs-loaded hyaluronan/collagen hydrogel (Zhang et al. 2021). The hydrogel was implanted in the rat hemi-transected spinal cord injury model under a 13 mT magnetic field. After 30 days of implantation, axonal regeneration was enhanced by the magnetoelectric effect with decreased glial expression and increased neuron formation. The combination of piezoelectric materials and magnetic stimulation introduces multilayered control in a cell- and location-specific manner to induce biological functions such as proliferation and differentiation (Li et al. 2021; Liu et al. 2021a; Zhang et al. 2021).

### Musculoskeletal applications

Magnetic hydrogels are ideal in enhancing cell differentiation and growth for musculoskeletal applications. A hydrogel was synthesized from type II collagen, hyaluronic acid, and polyethylene glycol, then combined with magnetic nanoparticles to create a magnetic hydrogel (MagGel) (Zhang et al. 2015). The magnetic nanoparticles were synthesized from poly(vinyl alcohol) (PVA)/FeC_3_l/FeCl_2_·4H_2_O mixed with ammonium hydroxide to form iron oxides. Cellular adhesion properties of the MagGel were tested using bone marrow derived mesenchymal stem cells (MSCs). No significant change in cell morphology was observed, but adhesion density was enhanced on MagGel. Nanoparticles were also detected in MSC cytoplasm. In another study, a hyaluronic acid-polyacrylic (HA-pAA) hydrogel was loaded with poly(lactic-co-glycolic acid) (PLGA) magnetic microcapsules (PPMMs) (Chiang et al. 2021). The hydrogel contained glutathione (GSH) and iron oxide nanoparticles (IO) arranged by layer-by-layer (LbL) method to form an LbL-PPMM/HA-pAA injectable magnetic material. The hydrogel could bind chondrocytes via CD44 receptors and be delivered to the damaged surface using an internal magnetic force. The LbL-PPMM/HA-pAA hydrogel along with chondrocytes were implanted in rabbits with damaged cartilage for a duration of 8 weeks. Chondrocytes within the LbL-PPMM/HA-pAA hydrogel were bound to the damaged sites through the magnetic force. The implantation site exhibited smooth and flat surface, indicating that the cartilage was repaired. The structure of repaired surface displaced a similar cell arrangement as normal cartilage tissue, suggesting that chondrocytes within the LbL-PPMM/HA-pAA hydrogel could be effectively oriented with a magnetic force to create a regular columnar array. Combining gelatin, β-cyclodextrin, and Fe_3_O_4_ created a magnetic gelatin/β-CD/Fe_3_O_4_ hydrogel (Huang et al. 2019). Cartilage specific markers that indicate MSC differentiation including COL2 and Aggrecan 2 were observed after 21-day culture. Hydrogel was injected into the knee joints of rabbits with articular cartilage damage and stimulated with pulse electromagnetic fields (PEMFs). Cartilage defects were found to be partially filled at 8 weeks and completely repaired by 12 weeks. The difference in time required for cartilage repair from these two studies could be due to the size of lesion as well as the composition of magnetic hydrogels. One delivered chondrocytes directly to the damage site, allowing them to bind with existing cartilage to form a smooth surface (Chiang et al. 2021), whereas the other stimulated the growth of new cartilage tissue using an external magnetic field (Huang et al. 2019). A previous study synthesized a biohybrid magnetically responsive microswimmer designed to stimulate skeletal muscle cells (Liu et al. 2022b). Microswimmers were synthesized by coating *C. pyrenoidosa,* in Fe_3_O_4_ nanoparticles to form a dense cover around the natural spherical shape of the microalgae. The *C. pyrenoidosa* and Fe_3_O_4_ microswimmers (CP@Fe_3_O_4_) were incubated with C2C12 cells. Near-infrared (NI)R laser radiation triggered the photothermal effects of the CP@Fe_3_O_4_ microswimmers on C2C12 cells, raising the temperature of myotubes to cause contraction. The CP@Fe_3_O_4_ microswimmers could also be guided to a specific location using a magnetic field of 2–8 Hz with the greatest increase in velocity. Upon reaching the exposed muscle of the anesthetized rat, microswimmers were stimulated with NIR radiation of 0.8 W/cm^2^, which increased muscle fiber temperature by 4.9 °C with contraction. The contraction frequency was linearly correlated to NIR radiation frequency. The excellent precision in stimulation at the microlevel demonstrated its potential applications for targeted therapy.

## Conclusions

Stimulation strategy via conductive, piezoelectric, or magnetic materials regulates cell functions such as proliferation and differentiation and activates important pathways involved in tissue development. It is important to recognize unique features associated with each stimulation method to design the optimal regenerative strategy for disease treatment. Direct electrical stimulation allows precise and controllable stimulus signals, which is ideal for studying electrically modulated biological response for in vitro study. Using direct electrical stimulation for in vivo disease treatment could be challenging given power requirements and special considerations for electrode-tissue interfaces (Liu et al. 2020c). Mechanically induced electrical stimulation from piezoelectric materials eliminates the need to rely on external power sources, allowing harvest of electrical energy from mechanical deformation directly from body’s natural movement such as blood vessel pulsation (Li et al. 2018). However, the lack of controlled mechanical stimulation applied to piezoelectric materials might result in insufficient electrical signals to produce substantial cellular changes for desired therapeutic benefits (D'Alessandro et al. 2021). Another major limitation associated with the clinical potential of piezoelectricity is the lack of biodegradability in most of the known materials with piezoelectric properties. For example, PVDF which is the most prevalently used piezoelectric material in research, is not biodegradable, limiting its potential applications for regenerative medicine. The poor recognition of interactions between tissues and piezoelectric materials is also an obstacle in applications of piezoelectric therapy (Wen and Liu 2014). Magnetic stimulation provides directional orientation and guidance for instructed regeneration, which is difficult to achieve with injectable solutions. Efforts are needed to design magnetic materials that can be precisely controlled to differentiate the healthy and affected tissues. As tissue regeneration is a complex process involving various cell types, it is important to understand fundamental biological mechanisms related to individual stimulation strategy on different cell types. Other active forms of electrical stimulation such as use of photovoltaics, specifically p-n and p-i-n junction diodes in semiconductors, are not discussed in this article. These designs require incorporation of metals and necessitate high power densities of light to generate sufficient photocurrents, which could potentially limit their therapeutic utility. Future development of stimulation strategies needs to be diverse its capabilities and properties that will allow us to address critical challenges in controlling specific biological response, reconstructing tissue and organ complexity for guided regeneration, and providing minimally invasive treatments for regenerative medicine.

## Data Availability

Not applicable.

## Abbreviations

ADSC: Adipose-derived mesenchymal stem cell
aNFC: Anionic nanofibrillar cellulose
ApoE: Apolipoprotein E
BaTiO3: Barium titanate
BNNT: Boron nitride nanotubes
CFO: Cobalt ferrite oxide
CHP: Cholesteryl-group-substituted pullulan
DCC: Deleted in Colorectal Cancer
DRG: Dorsal root ganglion
ECM: Extracellular matrix
EP: Electric pulse
Fe_3_O_4_: Iron (III) oxide
FWHM: Full width half maximum
GC: Glycol chitosan
GEL: Gelatin
GMNP: Gold magnetic nanoparticles
GSH: Glutathione
HA: Hyaluronic acid
IO/IONP: Iron oxide nanoparticles
KNN: Potassium-sodium niobate
LbL: Layer-by-layer
M.SNF: Magnetic short nanofiber
MagGel: Magnetic hydrogel
MAM: Magnetic alginate microparticles
MBP: Myelin basic protein
MCM41: Mesoporous silica nanoparticles
MHC: Myosin heavy chain
MLP: Muscle LIM protein
mPEG-PLV: Poly(ethylene glycol)-co-polyvaline
MPZ: Microscale piezoelectric zones
m-rGO: Responsive graphene oxide
MSC: Mesenchymal stem cells
MYH2: Myosin heavy chain (MHC)-IIa marker
nAK: Akermanite
NF 160: Neurofilament protein 160
NGF: Nerve growth factor
NPC: Neural progenitor cell
NSC: Neural stem cell
OE-MSC: Olfactory ecto-mesenchymal stem cell
PANI: Polyaniline
PCL: Polycaprolactone
PEDOT: Poly(3,4-ethylenedioxythiophene)
PEMF: Pulse electromagnetic field
PHB: Polyhydroxybutyrate
PHBV: Poly(3-hydroxybutyrate-co-3-hydroxyvalerate)
PLGA: Poly(lactide-co-glycolide)
PLLA: Poly(L-lactic acid)
PPMM: Poly(lactic-co-glycolic acid) (PLGA) magnetic microcapsules
PPy: Polypyrrole
PSD95: Postsynaptic density protein 95
PVA: Polyvinyl alcohol
PVDF: Polyvinylidene fluoride
PVDF-HFP: Polyvinylidene fluoride-hexafluoropropylene
PVDF-TrFE: Polyvinylidene fluoride-trifluoroethylene
PZT: Lead zirconate titanate
rGO: Reduced graphene oxide
SG: Silk fibroin/gelatin
SPION: Superparamagnetic iron oxide nanoparticles
STC2: Stanniocalcin 2
Trk: Tyrosine kinase
